# Thoracic ultrasound for the diagnosis of pneumonia in adults: a meta-analysis

**DOI:** 10.1186/s12931-015-0248-9

**Published:** 2015-07-25

**Authors:** Thomas Berlet

**Affiliations:** Inselspital/Bern University Hospital, Department of Intensive Care Medicine, 3010 Bern, Switzerland

## Letter to the Editor

**Thomas Berlet**

I read with interest the recent meta-analysis by Chavez and colleagues that was published in Respiratory Research, addressing the diagnostic performance characteristics of thoracic ultrasonography (TUS) in the diagnosis of pneumonia in adults. Favourable results for the diagnostic accuracy were reported, with calculated pooled sensitivities and specificities of 94 % and 96 %, respectively [1].

When I reviewed the original data that the meta-analysis by Chavez and colleagues was based upon, I detected a number of issues that I wish to comment on. The authors analysed ten studies [2–11]. Six of these were performed in patients who were admitted to emergency departments or medical wards and who presented with signs and symptoms suggestive of pneumonia [2, 6, 7, 9–11]. The study by Benci *et al.* enrolled 80 patients; 23 of these were diagnosed with interstitial pneumonia that could not be visualised with TUS [2]. The authors chose to exclude these patients from their calculation of diagnostic accuracy and focussed on the diagnosis of “alveolar pneumonia” instead. Therefore, the overall sensitivity of TUS for the diagnosis of any type of pneumonia was 61.7 %, and not 100 %, as cited by Chavez and colleagues [1]. The study by Parlamento and colleagues reported the sensitivity, but not the specificity, of TUS [6]. However, because normal TUS patterns were found in only 10 out of 17 patients who did not suffer from pneumonia, the specificity should have been calculated as 58.8 %, and not 100 %, as assumed by Chavez and colleagues [1]. The study by Reissig and colleagues enrolled 362 patients [9]. These authors chose to exclude equivocal TUS results from the calculation of diagnostic accuracy. By including these equivocal TUS findings in the calculation of diagnostic accuracy, sensitivity falls from 93.4 % to 92.1 %, and specificity from 97.7 % to 95.5 %. I believe that the data from the above-mentioned three studies should have been critically reviewed and adjusted prior to inclusion in the meta-analysis.

Chavez and colleagues included four studies that were performed in critically ill patients in intensive care units who suffered from a variety of respiratory conditions [3–5, 8]. I am concerned that Chavez and colleagues failed to appreciate the fact that three out of four of these studies investigated the diagnostic accuracy of TUS for alveolar consolidation of any aetiology, rather than for the diagnosis of pneumonia.

Lichtenstein and colleagues studied 32 acute respiratory distress syndrome patients; 22 of these were diagnosed with pneumonia [3]. In another study, Lichtenstein and colleagues investigated 60 patients [4]. The inclusion criterion in this study was “exploration of chest pain or severe thoracic disease”. Sixteen patients were diagnosed with pneumonia. While reporting high sensitivity and specificity of TUS for the detection of alveolar consolidation compared with computed tomography in both studies, Lichtenstein and colleagues did not differentiate between pneumonia patients and non-pneumonia patients. Xirouchaki and colleagues studied 42 patients [8]. While an admission diagnosis of sepsis or multiple organ dysfunction syndromes was present in 18 patients, no patient was diagnosed with pneumonia in their study. I believe that the results of these three studies should have been excluded from the meta-analysis because of methodological shortcomings.

A re-run of the meta-analysis by Chavez and colleagues [1], following correction of the data for sample size, sensitivity, and specificity in three studies [2, 6, 9], and exclusion of those studies that failed to involve [8] or clearly identify pneumonia patients [3, 4] was performed, using Meta-DiSc V1.4 software (Unidad de Biostadística, Hospital Universitario Ramón y Cajal, Madrid, Spain). This analysis yielded significantly lower results for sensitivity and specificity of TUS for pneumonia than the initial meta-analysis by Chavez and colleagues [1] (Table 1).

**Table 1:** Sensitivity and specificity table of thoracic ultrasonography for pneumonia

Study	Sample size	Sensitivity	95 % CI	Specificity	95 % CI
Benci et al. [2]	80	0.62	0.48-0.74	1.00	0.93-1.00
Lichtenstein et al. [5]	260	0.89	0.80-0.95	0.9	0.97
Parlamento et al. [6]	49	0.97	0.84-1.00	0.59	0.33-0.82
Cortellaro et al. [7]	120	0.99	0.94-1.00	0.95	0.83-0.99
Reissig et al. [9]	362	0.92	0.88-0.95	0.96	0.90-0.98
Testa et al. [10]	67	0.94	0.90-0.99	0.85	0.68-0.95
Unluer et al. [11]	72	0.96	0.82-1.00	0.84	0.7-0.94
**Pooled**	**1010**	**0.9**	**0.87-0.92**	**0.92**	**0.89-0.94**
Inconsistency I^2^		88.1 %		77.5 %	

CI is “confidence interval”

**Fig. 1:**
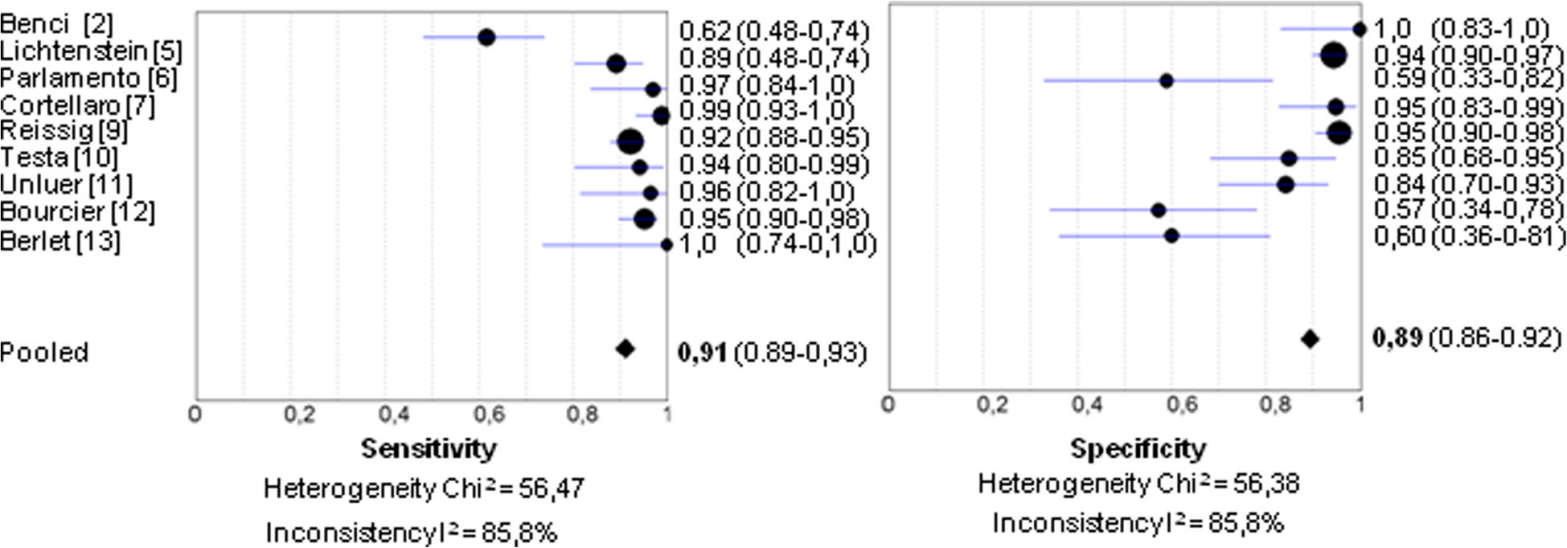
Forest plots for diagnostic accuracy of thoracic ultrasound for the diagnosis of pneumonia in adults

Since Chavez and colleagues performed their meta-analysis, another two clinical studies of the diagnostic accuracy of TUS for pneumonia have been published by Bourcier *et al.* [12], and Berlet *et al.* [13]. One-hundred forty-four [12], and 32 [13] patients were studied, raising the overall sample size to 1186. If the results of these studies are added to the meta-analysis, sensitivity and specificity for the diagnosis of pneumonia using TUS rises to 91 % (95 % confidence interval: 89–93 %) and specificity falls to 89 % (95 % confidence interval: 86–92 %). Heterogeneity between studies remains high, as reflected by high the inconsistency score. (Fig. 1).

## Conclusions

Re-analysis of the results of studies of the use of diagnostic ultrasonography for pneumonia confirms that TUS is a useful tool for the diagnosis of the inflammatory consolidation of pneumonia. However, further research is required to improve the diagnostic accuracy of TUS in the diagnosis of pneumonia in adults.

## Authors response

**Miguel A Chavez, Laura E Ellington, Robert H Gilman, William Checkley**

Over the last decade, there have been a growing number of research studies that evaluated the role of lung ultrasound for the diagnosis of pneumonia in adults [1]. Many of these studies preceded the development of standardized guidelines in sonographic methods and terminology [14], which may have contributed to heterogeneity in findings reported in our meta-analysis [1]. Differences in opinions about interpretation of findings may also affect how these studies are summarized. We appreciate Berlet’s concerns about potential differences between our analysis and his revised analysis, and take this opportunity to reiterate more explicitly our choices. Moreover, we also demonstrate that our choices or the addition of new information from recently published studies had an overall small effect on the estimates of diagnostic accuracy. First, Berlet states that we chose to exclude participants with interstitial pneumonia from our calculations in the study by Benci *et al.* [2]. However, given that Benci *et al.* did not include interstitial findings by lung ultrasound within their methods [2], we decided to include only participants with either normal or lobar pneumonia by CXR in our analysis [1].

Second, we agree with Berlet that the numbers contributed by the study of Parlamento *et al.* could be revised [6]. Specifically, Parlamento *et al.* found that 7 out of 17 participants who did not have pneumonia had an abnormal ultrasound. Of those, Parlamento *et al.* were able to rule out infectious causes of consolidation in two participants by examining the air bronchogram characteristics or by the absence of air bronchograms. Although Parlamento *et al.* did not explicitly discuss specific songraphic findings for the remaining five participants with alveolar-interstitial syndrome, and were implicitly considered as a negative ultrasound for pneumonia [6]. Assuming that those five participants were indeed false positives, the revised estimate of specificity would be 95 % (95 % CI 94 %-97 %).

Third, we agree with Berlet that equivocal ultrasound results (1.7 %) may affect estimates reported by Reissig *et al.* [9] and we could have reported this discrepancy in more detail. However, we did not consider this necessary since Reissig *et al.* [9] had already provided a detailed discussion about this point in their published paper. Moreover, although Reissig *et al.* had expert sonographers who performed the ultrasound, and in our own discussion we further emphasize that sonographer expertise is a critical element in assessing diagnostic accuracy.

Fourth, Berlet raises important concerns regarding studies performed in critically ill patients in intensive care units [3–5, 8] that were not directly addressed by our study [1]. Diagnostic accuracy in these studies was calculated for alveolar consolidation of any etiology including pneumonia, except for one study by Lichtenstein *et al.* [5] that specifically studied pneumonia. We agree with Berlet that the results of these studies [3, 4, 8] have some methodological shortcomings. In our systematic review, we presented subgroup analyses confirming that when we excluded all studies conducted in intensive care units, sensitivity was 95 % (95 % CI 93 %-97 %) and specificity was 94 % (95 % CI 91 %-97 %) for the remaining studies [2, 6, 7, 9–11].

**Fig. 2:**
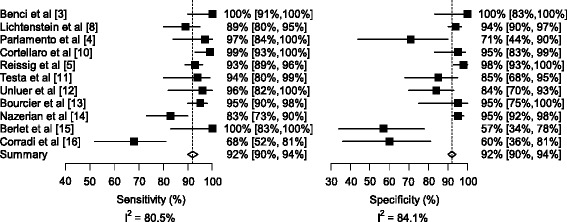
Revised forest plots for diagnostic accuracy of lung ultrasound for the diagnosis of pneumonia

Finally, since the publication of our meta-analysis, at least four new studies have been published [12, 13, 15, 16]. When we analyzed information from these recent studies [13, 15, 16], our revised estimates yielded an overall sensitivity of 92 % (95 % CI 90 %-94 %) and specificity of 92 % (95 % CI 90 %-94 %; Fig. 2), with an AUC of 0.97 (95 % CI, 0.95 to 0.99), which is similar in range to values reported by our original analyses [1]. None of the revised estimates, however, affects our final conclusion that lung ultrasound appears to be a reasonable alternative to chest radiography for the diagnosis of pneumonia in adults. However, we agree that further research is needed to assess the validity of lung ultrasound in a variety of settings and degrees of sonographer expertise.

## Abbreviations

CI: Confidence interval
TUS: Thoracic ultrasonography
