# Erratum to: Thoracic ultrasound for the diagnosis of pneumonia in adults: a meta-analysis

**DOI:** 10.1186/s12931-015-0259-6

**Published:** 2015-09-08

**Authors:** Thomas Berlet

**Affiliations:** Inselspital/Bern University Hospital, Department of Intensive Care Medicine, Bern, 3010 Switzerland

## Erratum

After publication of the original article [1], it came to the publisher’s attention that figure 2, associated with the Authors Response, had been accidently omitted. The original article has been corrected to include this figure. A citation to the figure has been inserted in the following sentence, “…our revised estimates yielded an overall sensitivity of 92 % (95 % CI 90 %-94 %) and specificity of 92 % (95 % CI 90 %-94 %; Fig. 2)…”
